# Lipocalin-2 released in response to cerebral ischaemia mediates reperfusion injury in mice

**DOI:** 10.1111/jcmm.12538

**Published:** 2015-02-20

**Authors:** Guona Wang, Yi-Chinn Weng, Xiqian Han, James D Whaley, Keith R McCrae, Wen-Hai Chou

**Affiliations:** a Department of Biological Sciences and School of Biomedical Sciences, Kent State UniversityKent, OH, USA; b Department of Cellular and Molecular Medicine and Taussig Cancer Institute, Cleveland ClinicCleveland, OH, USA

**Keywords:** lipocalin-2, reperfusion injury, neutrophil, biomarker, stroke

## Abstract

Thrombolysis remains the only effective therapy to reverse acute ischaemic stroke. However, delayed treatment may cause serious complications including hemorrhagic transformation and reperfusion injury. The level of lipocalin-2 (LCN2) is elevated in the plasma of ischaemic stroke patients, but its role in stroke is unknown. Here, we show that LCN2 was acutely induced in mice after ischaemic stroke and is an important mediator of reperfusion injury. Increased levels of LCN2 were observed in mouse serum as early as 1 hr after transient middle cerebral artery occlusion (tMCAO), reaching peak levels at 23 hrs. LCN2 was also detected in neutrophils infiltrating into the ipsilateral hemisphere, as well as a subset of astrocytes after tMCAO, but not in neurons and microglia. Stroke injury, neurological deficits and infiltration of immune cells were markedly diminished in LCN2 null mice after tMCAO, but not after permanent MCAO (pMCAO). *In vitro*, recombinant LCN2 protein induced apoptosis in primary cultured neurons in a dose-dependent manner. Our results demonstrate that LCN2 is a neurotoxic factor secreted rapidly in response to cerebral ischaemia, suggesting its potential usage as an early stroke biomarker and a novel therapeutic target to reduce stroke-reperfusion injury.

## Introduction

Ischaemic stroke is the most common neurological disease in the United States 1. Although ischaemia per se is responsible for the initiation of neuronal damage during stroke, reperfusion injury resulting from partial or complete recanalization may promote secondary infarct growth and worsen functional outcome 2. Reperfusion of oxygenated blood is followed by generation of reactive oxygen species (ROS) and a series of inflammatory events including activation and infiltration of circulating neutrophils, macrophages, and T-cells into infarcted brain tissue. Post-ischaemic inflammation is necessary for repair, but may have deleterious effects 3. Thus, identifying neuroprotective and neurotoxic components of post-ischaemic inflammation is central to developing effective and balanced therapeutic approaches to limit stroke-reperfusion injury.

Lipocalin-2 (LCN2), also known as 24p3 or neutrophil gelatinase-associated lipocalin (NGAL), was originally identified as a 25 kD protein secreted by activated neutrophils 4. Elevated plasma levels of LCN2 have been detected in patients suffering from ischaemic stroke 5,6. However, the role of LCN2 in stroke has not been defined. Molecular and cellular studies suggest that LCN2 is able to induce apoptosis in the mouse pro-B lymphocytic cell line FL5.12 7. LCN2 is internalized by its putative cell surface receptor, brain type organic cation transporter (BOCT). The internalized LCN2 chelates intracellular iron, thereby reducing intracellular iron concentrations and mediating apoptosis. On the basis of this mechanism, it has been suggested that LCN2 may induce neuronal apoptosis during stroke-reperfusion injury. Here, we report that LCN2 increases rapidly in response to cerebral ischaemia and promotes reperfusion injury in murine stroke. These results support the hypothesis that early detection and inhibition of LCN2 may prove useful in diagnosis and treatment of stroke-reperfusion injury.

## Materials and methods

### Ischaemic stroke model

Male *Lcn2*^*+/+*^ and *Lcn2*^*−/−*^ mice on a C57BL/6 background between 3 and 5 months of age were used for experiments 8. Focal cerebral ischaemia was induced by transient occlusion of the right MCA for 1 hr with a silicon-coated monofilament suture (Doccol, Sharon, MA, USA) 9. Twenty-three hours after the tMCAO, we determined oedema-corrected infarct volumes and brain swelling from coronal brain slices stained with 2,3,5-triphenyltetrazolium chloride (TTC, Sigma-Aldrich, St. Louis, MO, USA). All procedures were conducted in accordance with Institutional Animal Care and Use Committee policies.

### Regional cerebral blood flow

During focal cerebral ischaemia, regional cerebral blood flow (rCBF) was continuously monitored by Laser Doppler Flowmetry (Perimed, Ardmore, PA, USA). Mice were excluded from further studies if sufficient occlusion (<30% of the baseline) and reperfusion (>80% of the baseline) was not achieved, if excessive bleeding occurred during surgery, or if haemorrhage was found in the brain slices or at the base of the circle of Willis during post-mortem examination.

### Neurological deficit scores and corner tests

Twenty-three hours after the initiation of ischaemic stroke, mice were evaluated for neurological deficits using a four-tiered grading system 9. For the corner test, mice were placed between two vertical boards 10. The number of right (ipsilateral) turns when the mouse reached the wedge of the corner was recorded in ten trials.

### Collection of mouse serum and ELISA

At different time-points after ischaemic stroke, blood serum was collected for western blotting and ELISA. The level of LCN2 protein was quantified following the manufacturer’s protocol for mouse lipocalin-2/NGAL Quantikine ELISA kit (R&D, Minneapolis, MN, USA).

### Primary neuronal culture, oxygen glucose deprivation and MTT assays

Primary mixed neuronal-glial cultures were prepared from the cerebral cortex of post-natal day 1 (P1) to P3 mice as previously described 11. The culture was subjected to 1 hr of oxygen glucose deprivation (OGD) and 23 hrs of reoxygenation 12. The viability of cells after treatments was analysed by MTT assays according to the protocols for the Vybrant® MTT Cell Proliferation Assay Kit (Molecular Probes, Grand Island, NY, USA).

### Western blot analysis

Ipsilateral (I, right) and contralateral (C, left) hemispheres were isolated and homogenized using a Teflon-glass homogenizer as described 11. Neutrophils were isolated from mouse bone marrow by Percoll density gradient centrifugation 9. Proteins within brain homogenates, cell lysates and blood sera were separated by Bis-Tris gels (Invitrogen, Grand Island, NY, USA), and analysed by western blotting. Immunoreactive bands were detected using enhanced ECL (Pierce, Rockford, IL, USA), imaged using a Luminescent Image Analyzer LAS-3000 (Fujifilm, Edison, NJ, USA), and quantified by NIH ImageJ.

### Immunofluorescence microscopy

Primary cultured neurons and brain sections were fixed with 4% paraformaldehyde and immunostained as described 11. The images were acquired using an Olympus (Center Valley, PA, USA) FV500/IX81 confocal microscope.

### Statistical analysis

Quantitative data were expressed as mean ± SEM and analysed using Prism 5.0 (GraphPad, La Jolla, CA, USA). One or two-tailed unpaired *t*-tests or one-way anova with Newman–Keuls *post hoc* tests were employed to determine statistical significance between means. The values of *P* < 0.05 in all tests were considered to be statistically significant.

The methods are described in detail in the Data S1.

## Results

### Acute induction of LCN2 in mouse serum after ischaemic stroke

**Figure 1:**
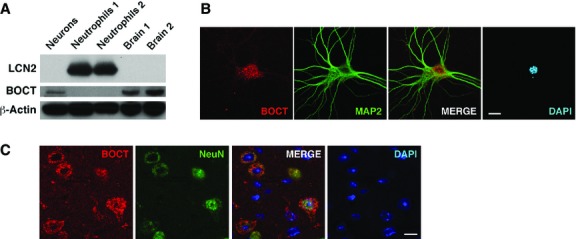
Expression of LCN2 and BOCT under non-stimulated conditions. (A) A representative Western blot shows the expression of LCN2 (∽25 kD) and BOCT (∽65 kD) in cultured cortical neurons (neurons), neutrophils and brain homogenates respectively. Samples were normalized to β-Actin. Confocal images show the expression pattern of BOCT in cultured cortical neurons (B) and brain sections (C). Neurons were stained for BOCT (red) and neuronal marker MAP2 or NeuN (green). Nuclei were stained with DAPI (blue). Scale bars, 10 μm.

We first assessed the expression of LCN2 and its putative receptor BOCT in neutrophils and neurons under non-ischaemic conditions. LCN2 protein was enriched in neutrophils, but undetectable in neuronal cells (Fig.1A). In contrast, BOCT protein was detected in cultured neurons and brain tissues, but not neutrophils. Immunofluorescence staining revealed a dense, punctate pattern of BOCT expression in microtubule-associated protein 2 (MAP2)-positive cultured neurons (Fig.1B) as well as neuronal nuclear protein (NeuN)-positive neurons in cerebral cortex (Fig.1C).

**Figure 2:**
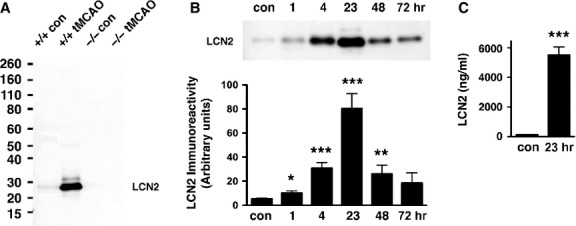
Induction of LCN2 in mouse serum after ischaemic stroke. (A) Mouse sera collected from *Lcn2*^*+/+*^ and *Lcn2*^*−/−*^ mice after 1 hr of tMCAO and 23 hrs of reperfusion were analysed by western blotting using anti-LCN2 antibody. The serum of mice without tMCAO was collected as a control (con). (B) Mouse sera collected at different time-points after 1 hr of tMCAO were analysed by western blotting using anti-LCN2 antibody. The top panel is a representative Western blot. The LCN2 protein bands were quantified by densitometry in the bottom panel (*n* = 3–6). There was a significant induction of LCN2 at 1 hr (*P < 0.05), 4 and 23 hrs (***P < 0.0005) and 48 hrs (**P < 0.005) after tMCAO compared with control (two-tailed, unpaired *t*-test). (C) Levels of LCN2 in control mouse sera and at 23 hrs after tMCAO were determined by ELISA (*n* = 7). ***P < 0.0001 compared with control (two-tailed, unpaired *t*-test).

Previous studies demonstrated elevated levels of LCN2 in human plasma 1–3 days after ischaemic stroke 5,6. To assess the time course of LCN2 induction after ischaemic stroke, we occluded the MCA for 1 hr and collected sera of wild-type (WT) and *Lcn2*^*−/−*^ mice at 23 hrs after reperfusion 9. A major immunoreactive band of LCN2 (∽25 kD) was detected in the sera of WT, but not *Lcn2*^*−/−*^ mice (Fig.2A). The expression of LCN2 was barely detectable in the control sera of WT mice not subjected to tMCAO. To more carefully examine the temporal profile of LCN2 induction, we analysed sera at different intervals after tMCAO (Fig.2B). Increased LCN2 levels were observed 1 hr after tMCAO, continued to rise between 4 and 23 hrs, and were diminished by 48–72 hrs. The level of LCN2 protein was 133.1 ± 18.3 ng/ml (6.4 nmol/L) in control mouse sera, comparable with previous studies 8, but increased 41-fold to 5509 ± 539.5 ng/ml (263.904 nmol/L) 23 hrs after tMCAO (Fig.2C).

### Detection of LCN2 in the infiltrated neutrophils and astrocytes

**Figure 3:**
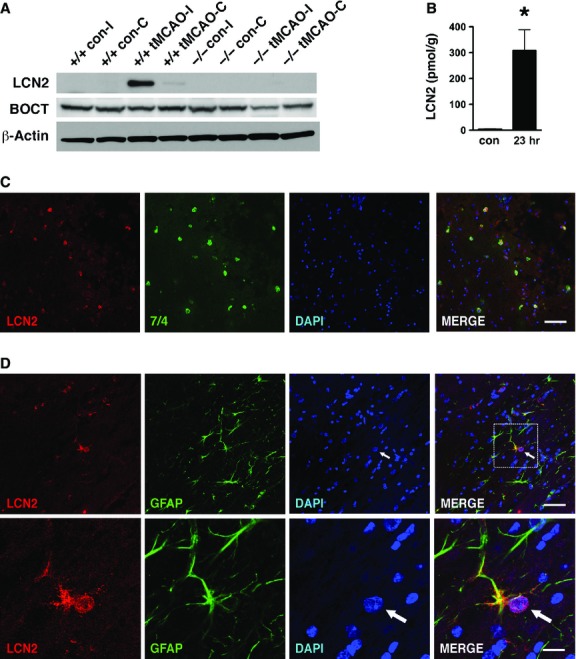
Detection of LCN2 in infiltrating neutrophils and astrocytes in the ipsilateral hemisphere after tMCAO. (A) The ipsilateral (I) and contralateral (C) hemispheres of *Lcn2*^*+/+*^ and *Lcn2*^*−/−*^ mice were isolated after 1 hr of tMCAO and 23 hrs of reperfusion. The hemispheres of mice without tMCAO were used as controls (con). The brain homogenates were analysed by western blotting using anti-LCN2 and anti-BOCT antibodies. β-Actin was used as a loading control. (B) The levels of LCN2 in the brain homogenates of ipsilateral hemispheres at 23 hrs after tMCAO were determined by ELISA. The ipsilateral hemispheres of mice without tMCAO were prepared as controls (con). *P < 0.05 compared with control (two-tailed, unpaired *t*-test). (C) Immunoreactivities of LCN2 (red) and neutrophil-specific marker 7/4 (green) in the ipsilateral hemispheres at 23 hrs after tMCAO were detected using confocal microscopy. (D) Confocal images of LCN2 (red) and GFAP-positive astrocytes (green) in the ipsilateral hemispheres at 23 hrs after tMCAO. Amplified images of LCN2- and GFAP- positive astrocytes are shown in the bottom panels. The arrows indicate the enlarged and eccentric nuclei of GFAP-positive astrocytes. Merged images indicate colocalization of LCN2 with 7/4 or GFAP in yellow. Scale bars, 50 μm (C and D top panel) and 12.5 μm (D bottom panel).

To determine whether LCN2 is induced in brain tissues after ischaemic stroke, we analysed brain homogenates of WT and *Lcn2*^*−/−*^ mice before and after tMCAO (Fig.3A). LCN2 protein was undetectable in the uninjured brains of WT and *Lcn2*^*−/−*^ mice, but elevated in the ipsilateral (I), and faintly in the contralateral (C) hemispheres 23 hrs after tMCAO (Fig.3A). The LCN2 level measured by ELISA was 3.9 ± 1.3 pmol/g (picomoles per gram of brain proteins) in the control brain tissues and reached 307.3 ± 81.7 pmol/g in the ipsilateral brain tissues 23 hrs after tMCAO (Fig.3B), representing an 80-fold increase in expression. The expression level of BOCT receptor in brain homogenates was unchanged after tMCAO, and in the absence of LCN2 (Fig.3A).

**Figure 4:**
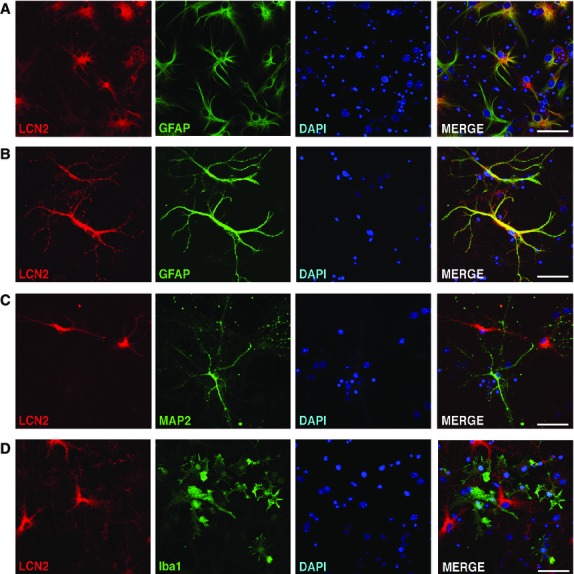
Expression of LCN2 in the subset of astrocytes after the OGD. (A and B) Confocal images show the expression of LCN2 (red) and GFAP (green) in cultured cortical neurons at 23 hrs after 1 hr of OGD. Merged images indicate colocalization of LCN2 with GFAP in yellow. Images of LCN2-positive cells and MAP2-positive neurons (C) and Iba1-positive microglia (D) after the OGD demonstrating no colocalization. Scale bars, 50 μm.

We next assessed the cellular localization of LCN2 in the ipsilateral hemispheres. Brain sections isolated 23 hrs after tMCAO were stained with antibodies recognizing LCN2 and specific markers for neutrophils (7/4), neurons (NeuN), microglia (Iba1) or astrocytes (GFAP). LCN2 was present in the 7/4-positive, blood-derived neutrophils surrounding striatal lesions (peri-infarct or penumbra area) in the ipsilateral hemisphere (Fig.3C). LCN2 expression was also detected in a subset of GFAP-positive astrocytes in the ipsilateral hemisphere (Fig.3D), but not in NeuN-positive neurons or Iba1-positive microglia (data not shown). To confirm the cellular distribution of LCN2, we subjected primary mixed neuronal-glial cultures to OGD, an *in vitro* model of ischaemic stroke. After 1 hr of OGD and 23 hrs of reoxygenation, LCN2 was present in a subset of the GFAP-positive astrocytes, but absent in MAP2-positive neurons and Iba1-positive microglia (Fig.4). Some of the LCN2-positive astrocytes displayed morphological characteristics of reactive astrocytes with enlarged nuclei and hypertrophy of cell bodies and processes (Fig.3D and 4B) 13.

### Reduced brain injury in LCN2 null mice after transient, but not permanent, MCAO

**Figure 5:**
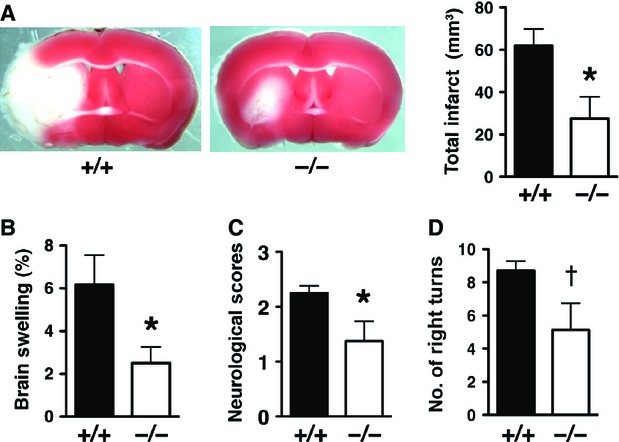
Stroke injury is reduced in LCN2 null mice after tMCAO. (A) Representative images of TTC-stained brain slices from *Lcn2*^*+/+*^ and *Lcn2*^*−/−*^ mice after 1 hr of tMCAO and 23 hrs of reperfusion. Viable tissue is stained in red colour, while the infarcted area in white colour. Total Infarct volume (A), brain swelling (B), neurological deficit scores (C) and corner tests (D) were determined after 1 hr of tMCAO and 23 hrs of reperfusion from *Lcn2*^*+/+*^ (*n* = 8) and *Lcn2*^*−/−*^ (*n* = 8) mice. *P < 0.05 compared with WT mice (two-tailed, unpaired *t*-test). ^†^*P* < 0.05 compared with WT mice (one-tailed, unpaired *t*-test).

To assess the effect of stroke-induced LCN2 *in vivo*, we subjected WT and *Lcn2*^*−/−*^ mice to tMCAO with 1 hr of ischaemia followed by 23 hrs of reperfusion (Fig.5). Infarct volume was reduced by ∽56% in *Lcn2*^*−/−*^ mice compared with WT mice (62.05 ± 7.88 mm^3^ for WT *versus* 27.47 ± 10.29 mm^3^ for *Lcn2*^*−/−*^; *P* < 0.05; Fig.5A). Likewise, brain swelling in the ipsilateral hemispheres was reduced by ∽60% in *Lcn2*^*−/−*^ mice (6.2% ± 1.4% for WT *versus* 2.5% ± 0.8% for *Lcn2*^*−/−*^; *P* < 0.05; Fig.5B). Reduction in cerebral infarct size and oedema in *Lcn2*^*−/−*^ mice was reflected in their functional outcome. *Lcn2*^*−/−*^ mice showed improved neurological outcome (*P* < 0.05; Fig.5C) and reduced sensorimotor asymmetry in corner tests (*P* < 0.05; Fig.5D) compared to WT controls.

At baseline, *Lcn2*^*−/−*^ mice display normal motor and sensory function and coordination 8,14, and comparison of brains from WT and *Lcn2*^*−/−*^ mice showed no gross differences in size or anatomy (Fig. S1). To determine whether the attenuated brain damage in *Lcn2*^*−/−*^ mice was because of changes in cerebral vasculature, we examined the distribution of cerebral vessels by ink perfusion. No difference was observed in the origins of the MCA or other major blood vessels of the circle of Willis (Fig. S1A), the degree of posterior communicating artery (PcomA) patency (Fig. S1A and B), and the size of MCA territory (Fig. S1C and D). In addition, the rCBF in the MCA area monitored by Laser Doppler Flowmetry (Fig. S1E) was similar between WT and *Lcn2*^*−/−*^ mice before and during ischaemia, and after reperfusion (Fig. S1F).

Since LCN2 was induced in both blood and brain tissues after tMCAO, we evaluated the contribution of LCN2 from ischaemic brain by minimizing the input of circulating blood using a permanent MCAO (pMCAO) model in which the MCA was occluded for 24 hrs without reperfusion (Fig. S2) 2,15. Infarct volume (55.58 ± 9.69 mm^3^ for WT *versus* 66.95 ± 13.06 mm^3^ for *Lcn2*^*−/−*^; *P* > 0.05; Fig. S2A and B) and brain swelling of the ipsilateral hemispheres (5.95% ± 1.06% for WT *versus* 7.56% ± 1.99% for *Lcn2*^*−/−*^; *P* > 0.05; Fig. S2C) was marginally increased in *Lcn2*^*−/−*^ mice after pMCAO. The neurological scores after pMCAO were slightly worse in *Lcn2*^*−/−*^ mice (*P* > 0.05; Fig. S2D). The reduced stroke injury in LCN2 null mice observed in the tMCAO, but not in the pMCAO model, suggests that LCN2 is an essential factor mediating reperfusion injury after ischaemic stroke.

### LCN2 mediated immune cell recruitment and neuronal apoptosis

**Figure 6:**
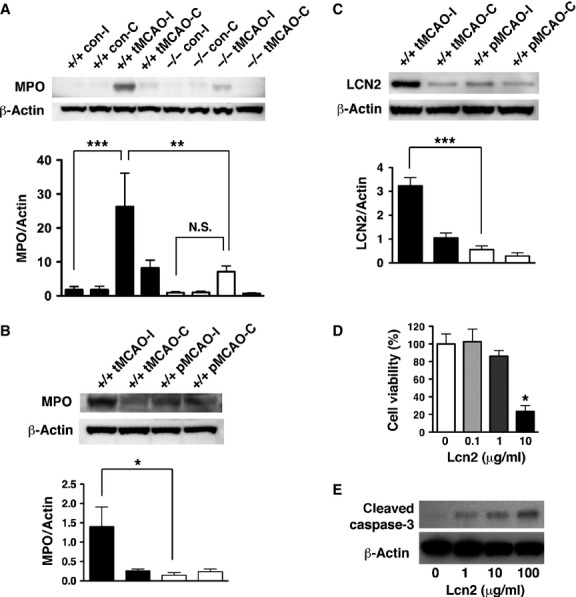
The effect of LCN2 on the infiltration of peripheral immune cells and the survival of neuronal cells. (A) Immune cells infiltration was analysed by the levels of MPO in brain homogenates. The ipsilateral (I) and contralateral (C) hemispheres of *Lcn2*^*+/+*^ (*n* = 4) and *Lcn2*^*−/−*^ (*n* = 4) mice were collected at 23 hrs after 1 hr of tMCAO. The brain homogenates were prepared and analysed by western blotting using antibodies against MPO. β-Actin served as a loading control. Representative Western blots show the expression of MPO heavy chain (∽55 kD) in brain homogenates. MPO immunoreactivity normalized to β-Actin (MPO/Actin) was compared between means using one-way anova with Newman–Keuls *post hoc* tests. There was a significant induction of MPO after tMCAO (***P < 0.0001) compared with controls, and a significant reduction in MPO induction in the ipsilateral hemispheres of *Lcn2*^*−/−*^ mice (**P < 0.01). Immune cell infiltration (B) and LCN2 induction (C) in brains after tMCAO and pMCAO were analysed by western blotting using antibodies against MPO and LCN2, respectively. MPO or LCN2 immunoreactivity normalized to β-Actin (MPO/Actin or LCN2/Actin) was compared between means using one-way anova with Newman–Keuls *post hoc* tests. There was a significant reduction in MPO (*P < 0.05) and LCN2 (***P < 0.0001) levels in the ipsilateral hemispheres after pMCAO compared with tMCAO. (D and E) Primary neurons were incubated with different concentrations of recombinant human LCN2 protein at 37°C for 24 hrs (*n* = 3). (D) The viability of neurons after the LCN2 treatments was determined by MTT assays. *P < 0.05 compared with treatments with PBS vehicle (two-tailed, unpaired *t*-test). (E) Induction of apoptosis after the LCN2 treatments was determined by western blotting using antibodies against cleaved caspase-3 (Asp175) (19 kD). β-Actin was used as a loading control.

Peripheral immune cells infiltrate ischaemic brain tissue during reperfusion and exacerbate brain injury 2,9. Since LCN2 was detected in infiltrating neutrophils after tMCAO (Fig.3C), we determined whether LCN2 deficiency affects the infiltration of circulating immune cells. The presence of infiltrating immune cells in brain homogenates of WT and *Lcn2*^*−/−*^ mice were detected by western blotting using anti-myeloperoxidase (MPO) antibody. MPO is a key enzyme expressed in blood-borne neutrophils, macrophages and monocytes, and has been used as a marker for inflammation after stroke 16. Significantly increased amounts of MPO were present after tMCAO in the ipsilateral hemispheres of WT, but not *Lcn2*^*−/−*^ mice (Fig.6A), demonstrating that LCN2 deficiency limited the infiltration of immune cells into ischaemic brain. The stroke-induced MPO (Fig.6B) and LCN2 (Fig.6C) was minimally detected in the pMCAO model and significantly lower than the tMCAO model, showing that the infiltration of immune cells in pMCAO was not as pronounced as in tMCAO.

Infiltrating immune cells (*e.g*. neutrophils) release free radicals and proteins that aggravate brain injury after reperfusion 2,9. LCN2 was originally identified as a secreted protein from neutrophils 4, thus it is possible that LCN2 promotes neuronal injury in the tMCAO model. To determine whether LCN2 directly mediates neuronal cell death, we treated cultured neurons with increasing concentrations of recombinant LCN2. Addition of LCN2 reduced cell viability (Fig.6D) and induced apoptotic cell death (Fig.6E) in a concentration-dependent manner.

## Discussion

Our results identify LCN2 as an acute-phase protein that mediates reperfusion injury after ischaemic stroke in mice. The elevated level of LCN2 after stroke may exacerbate cerebral infarction and contribute to poor neurological outcome by enhancing inflammatory cell infiltration and promoting neuronal apoptosis. We detected the induction of LCN2 in mouse serum within 1 hr after tMCAO (Fig.2), suggesting the possibility of using LCN2 as an early blood biomarker of stroke. Nearly 95% of stroke patients are unable to benefit from thrombolytic therapy because they are diagnosed beyond a critical 4.5-hr time period following the onset of symptoms 17. Development of rapid diagnostic assays using LCN2 may facilitate early diagnosis, reduce the risk of cerebral haemorrhage and increase the overall efficacy of thrombolytic therapy 18.

**Figure 7:**
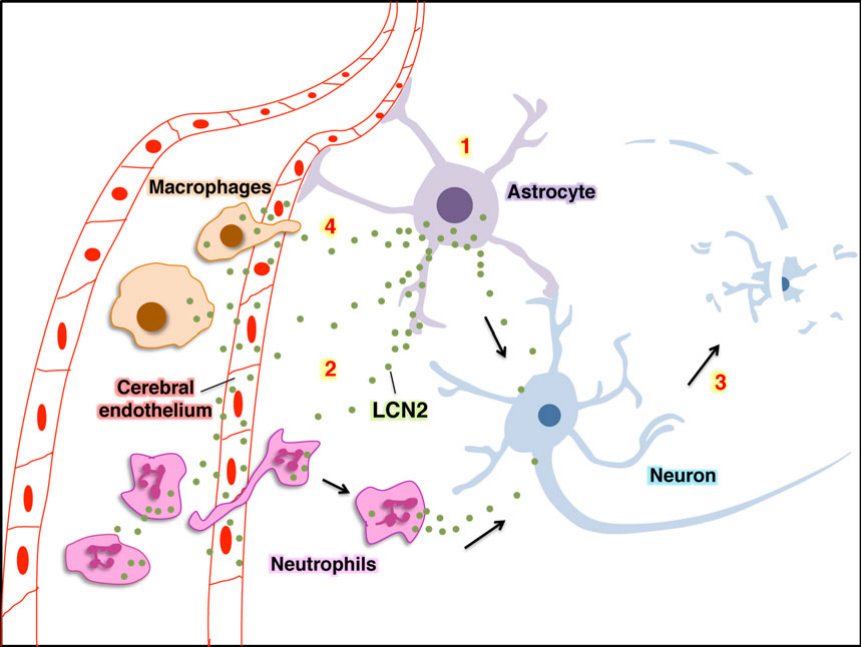
A proposed model for the sequence of LCN2 induction and its effect on neurons. (1) LCN2 is induced and released from astrocytes in response to stroke-reperfusion injury. (2) The released LCN2 may cause the induction and release of LCN2 from cerebral endothelial cells and neutrophils, and stimulate the infiltration of circulating neutrophils. (3) LCN2 released from astrocytes and neutrophils may cause additional neuronal cell death and (4) recruit circulating macrophages.

Expression of LCN2 after stroke was increased not only in sera but also in brain, where it was localized to infiltrating neutrophils and a subset of astrocytes (Fig.3 and 4). A recent study confirmed the induction of LCN2 in astrocytes as well as cerebral endothelium after tMCAO 19. Minutes after ischaemic stroke, resident brain cells including astrocytes release ROS, proinflammatory cytokines and chemokines, thus inducing the expression of adhesion molecules on cerebral endothelium and promoting transendothelial migration of circulating neutrophils 2. Infiltrating neutrophils release ROS as well as cytokines and chemokines that further amplify the brain-inflammatory response. It is likely that LCN2 from astrocytes initiates the neuroinflammatory event, causing release of LCN2 from cerebral endothelium and circulating neutrophils (Fig.7). The astrocyte-endothelial interactions have been well documented in the blood-brain barrier (BBB) 20. Several factors secreted by astrocytes such as transforming growth factor-beta, src-suppressed C-kinase substrate, and glial-derived neurotrophic factor affect the gene expression of endothelial cells and the functions of BBB 21–23. LCN2 expression can be up-regulated by ROS 24, thus ROS released by reactive astrocytes and infiltrating neutrophils may also contribute to the induction of LCN2 in endothelium. Moreover, LCN2 released from astrocytes and infiltrating neutrophils may cause additional neuronal damage after ischaemic stroke (Fig.7). A recent study 25 supports our finding that recombinant LCN2 induced cell death in primary neurons (Fig.6), but not in astrocytes, microglia and oligodendrocytes. These results suggest that LCN2 is selectively toxic to neurons, but not neuroglia.

Our finding that the infiltration of MPO-positive immune cells is reduced in LCN2 null mice after tMCAO is consistent with recent reports focused on LCN2 function in other models. For example, immune cell recruitment to the site of injury in LCN2 null mice is diminished in mouse models of spinal cord injury 14 and heterotopic heart transplantation 26. Neutrophils isolated from LCN2 null mice display defective chemotaxis *in vitro* 27. Moreover, recombinant LCN2 stimulates migration of neutrophils *in vitro* and *in vivo* 28. These results suggest that LCN2 is a paracrine chemoattractant for immune cells during ischaemia-reperfusion injury. Similar pro-neuroinflammatory effects have been observed for S100A8 and S100A9, two members in the S100 family of calcium-binding proteins 29. S100A8 and S100A9, also known as myeloid-related proteins 8 and 14, are abundantly expressed in neutrophils and stored in the secondary granules where LCN2 is detected 4. These two S100 proteins have been characterized as endogenous ligands of toll-like receptor 4 30, and stimulate neutrophil chemotaxis and transmigration 31. S100A8 and S100A9 are induced in ischaemic hemispheres after tMCAO and deficiency of these proteins reduces the accumulation of inflammatory cells as well as cerebral swelling and infarction in post-ischaemic brain 32.

The plasma concentration of LCN2 is increased in patients after ischaemic stroke 5,6. During a 4-year follow-up, stroke patients with higher levels of LCN2 had higher cardiovascular mortality 6. These results together with the findings in this report suggest that LCN2 has detrimental effects following stroke. This raises the intriguing possibility that LCN2 inhibitors or anti-LCN2 antibodies could be important therapeutic candidates to modulate post-ischaemic inflammation in patients with ischaemic stroke.

## Supporting Information

**Data S1** Assessment of cerebrovascular anatomy and determination of regional cerebral blood flow (rCBF). **Data S2** Infarct size is similar in WT and LCN2 null mice after pMCAO.
