# Supplementary material for: Lipocalin-2 released in response to cerebral ischaemia mediates reperfusion injury in mice
Source: J Cell Mol Med. 2015 Feb 20;19(7):1637–45. doi: 10.1111/jcmm.12538 (PMC4511361; doi:10.1111/jcmm.12538)
Supplement: Supplementary file 1 [file jcmm0019-1637-sd1.pdf]

Supplemental Information for

Lipocalin-2 released in response to cerebral ischemia mediates reperfusion injury in mice

Guona Wang[a], Yi-Chinn Weng[a], Xiqian Han[a], James D. Whaley[a], Keith R. McCrae[b], Wen-Hai Chou[a,] *

[a] Department of Biological Sciences and School of Biomedical Sciences, Kent State University, Kent, Ohio, USA

[b] Department of Cellular and Molecular Medicine and Taussig Cancer Institute, Cleveland Clinic, Cleveland, Ohio, USA

*Correspondence to: Wen-Hai Chou, Ph.D., Department of Biological Sciences, Kent State University, Kent, Ohio 44242, USA; Tel.: 330-672-2979, Fax: 330-672-3713, E-mail: wchou2@kent.edu

Supplemental Methods

Animal

Male *Lcn2*$^{+/+}$ and *Lcn2*$^{-/-}$ mice on a C57BL/6 background between 3 and 5 months of age were used for all experiments [1]. Mice were genotyped by PCR of tail DNA. All procedures were conducted in accordance with Institutional Animal Care and Use Committee policies.

Middle cerebral artery occlusion (MCAO)

Focal cerebral ischemia was induced by transient or permanent intraluminal monofilament occlusion of the right MCA [2-4]. Mice weighing 25-35 g were anesthetized with 1.5% isoflurane in 30% O_2 / 70% N_2 using the V-10 Anesthesia system (VetEquip). Rectal temperature was monitored and maintained at 37 ± 0.5 °C throughout the procedure by the TR-200 homeothermic temperature system (Fine Science Tools). A silicon-coated monofilament suture (Doccol) was inserted into the lumen of the external carotid artery and passed into the internal carotid artery (ICA) 9-10 mm past the bifurcation of the common carotid artery (CCA), resulting in occlusion of the MCA. Following one hour of MCAO, the suture was removed from the CCA to induce reperfusion for the tMCAO model. The occluding suture was left in place for 24 hours until sacrificing the mice for the pMCAO model.

Regional cerebral blood flow (rCBF)

During focal cerebral ischemia, rCBF was continuously monitored by Laser Doppler Flowmetry (PF5001, Perimed) with a flexible 0.8-mm fiber optic extension probe (Probe 407, Perimed). The tip of the probe was affixed to the intact skull over the right cortex at 2 mm posterior to the bregma and 4 mm lateral to the midline. The rCBF was recorded 20 minutes before ischemia to obtain a steady-state baseline (100%) and continued until 15 minutes after the reperfusion. The percentage of the baseline rCBF (%) was calculated as the percentage relative to the baseline. The abrupt drop of Laser Doppler signal to ~20% of the baseline rCBF indicated the occlusion of MCA by the suture. Mice were excluded from further studies if sufficient occlusion (<30% of the baseline) and reperfusion (>80% of the baseline) was not achieved based on the Laser

Doppler Flowmetry, if excessive bleeding occurred during surgery, or if hemorrhage was found in the brain slices or at the base of the circle of Willis during postmortem examination.

Neurological deficit scores and corner tests

Twenty-three hours after the initiation of ischemic stroke, mice were evaluated for neurological deficits using a four-tiered grading system [3,5]. Score 0 indicates no observed neurological deficit; score 1, inability to walk straight; score 2, circling toward the paretic side; score 3, falling on the paretic side; and score 4, loss of the righting reflex. For the corner test, mice were placed between two vertical boards attached at a 30° angle [6]. The number of right (ipsilateral) turns when the mouse reached the wedge of the corner was recorded in ten trials.

Collection of mouse serum

At different time points after ischemic stroke, mice were anesthetized with 5% isoflurane and euthanized by cervical dislocation. The blood was collected from the decapitated trunk and placed at room temperature for one hour. The blood was centrifuged at 2000 X g for 20 min at room temperature, and the supernatant was collected as blood serum for western blotting and ELISA [7].

Determination of infarct volume and brain swelling

Twenty-three hours after one hour of tMCAO, mice were anesthetized with 5% isoflurane and euthanized by cervical dislocation. The brain was removed and sliced into 1-mm-thick coronal sections using a brain matrix (Braintree Scientific) on ice. The brain sections were stained with 2% 2,3,5-triphenyltetrazolium chloride (TTC, Sigma-Aldrich) in 1X PBS at room temperature for 20 minutes and fixed in 10% formalin (Sigma-Aldrich) at 4 °C until imaging. Both sides of sections were photographed using a Leica EZ4HD stereomicroscope with integrated High Definition digital camera. The areas of cerebral infarction and of hemispheres were measured using the NIH ImageJ by an investigator blinded to the treatment conditions. The total infarct volumes were calculated using the following equations to consider the effect of edema: infarct area on one side of brain section = area of contralateral hemisphere – (area of ipsilateral hemisphere – area of infarct area) [8,9]. Total infarct volume = (front infarct area of a section + rear infarct area of the same section) / 2 × thickness of the section × total numbers of sections. Brain swelling (the amount of edema formation) = [(ipsilateral hemisphere volume – contralateral hemisphere volume) / contralateral hemisphere volume] × 100.

Primary neuronal culture and oxygen glucose deprivation (OGD)

Primary mixed neuronal-glial cultures were prepared from the cerebral cortex or hippocampus of postnatal day 1 (P1) to P3 mice as previously described [10-12]. Primary neuronal-glial cells were cultured in a humidified CO_2 incubator with 5% CO_2 and 95% air at 37 °C for 10-14 days *in vitro* (DIV 10-14). To initiate OGD, culture media were changed into Neurobasal-A media without glucose (Invitrogen) containing B27 Supplement Minus AO (antioxidants), Glutamax, and sodium pyruvate [13]. The cultures were placed into a humidified Modular Incubator Chamber (Billups-Rothenberg) and flushed with 5% CO_2 and 95% N_2 for 5 min. The chamber was then sealed and incubated at 37 °C for one hour of OGD. To mimic reoxygenation, the culture was removed from the Modular Incubator Chamber and the media replaced with regular Neurobasal-A media with glucose and incubated with 5% CO_2 and 95% air at 37 °C for 23 hours.

Western blot analysis
Mice were anesthetized at specified time points after ischemia by isoflurane inhalation and perfused intracardially with saline. Ipsilateral (I, right) and contralateral (C, left) hemispheres were isolated and homogenized using a Teflon-glass homogenizer as described [11,12,14]. Neutrophils were isolated from mouse bone marrow by Percoll density gradient centrifugation [3,15]. Proteins within brain homogenates, cell lysates and blood sera were separated by NuPAGE 4–12% Bis-Tris gels (Invitrogen), then transferred to PVDF membranes and analyzed by western blotting using goat anti-LCN2 (1:1000, R&D Systems, Cat# AF1857, RRID: AB_355022), goat anti-BOCT (1:500, ProSci, Cat# 46-899, RRID: AB_1948680), mouse anti-β-Actin (1:2000, Sigma-Aldrich, Cat# A4700, RRID: AB_476730), rabbit anti-cleaved caspase-3 (Asp175) (1:1000, Cell Signaling, Cat# #9661, RRID: AB_331440), and goat anti-myeloperoxidase (MPO) heavy chain (1:400, R&D, Cat# AF3667, RRID: AB_2250866) antibodies. Immunoreactive bands were detected using enhanced chemiluminescence (ECL) (Pierce), imaged using a Luminescent Image Analyzer LAS-3000 (Fujifilm), and quantified by NIH ImageJ.

Immunofluorescence microscopy
Primary cultured neurons were fixed with 4% paraformaldehyde (PFA) in PBS and immunostained as described [11,12]. Mice were perfused intracardially with 4% PFA, and coronal sections (50 μm) of fixed brains were prepared using a Leica CM1950 Cryostat [3,11]. Fixed neurons or brain sections were incubated overnight at 4 °C with primary antibodies using the following concentrations: goat anti-LCN2 (1:100, R&D, Cat# AF1857, RRID: AB_355022), goat anti-BOCT (1:100, ProSci, Cat# 46-899, RRID: AB_1948680), rat anti-mouse neutrophil mAb - clone 7/4 (1:200, Serotec, Cat# MCA771GA, RRID: AB_324243), rabbit anti-GFAP (1:100, Abcam, Cat# ab48050, RRID: AB_941765), rabbit anti-Iba1 (1:100, Wako Chemicals, Cat# 019-19741, RRID: AB_839504), mouse anti-MAP2 (1:200, Millipore, Cat# MAB3418, RRID: AB_11212326) and mouse anti-NeuN (1:100, Millipore, Cat# MAB377, RRID: AB_11210778) antibodies. After washing, the neurons and sections were stained with Alexa Fluor conjugated secondary antibodies (1:200, Jackson ImmunoResearch, Donkey anti-Goat IgG, Cat# 705-035-147, RRID: AB_2313587; Donkey anti-Rabbit IgG, Cat# 711-035-152, RRID: AB_10015282; Donkey anti-Mouse IgG, Cat# 715-035-150, RRID: AB_2313608) and mounted in media containing DAPI (4', 6-diamidino-2-phenylindole) (Vector Laboratories). The images were acquired using an Olympus FV500/IX81 confocal microscope (Olympus).

ELISA
The level of LCN2 protein in mouse sera and brain homogenates was quantified following the manufacturer's protocol for mouse lipocalin-2/NGAL Quantikine ELISA kit (R&D).

MTT assays
The viability of cells after treatment with different concentrations of recombinant human LCN2 protein (R&D) was analyzed by MTT assays according to the protocols for the Vybrant® MTT Cell Proliferation Assay Kit (Molecular Probes). Briefly, cells were incubated with 1.2 mM MTT (3-(4,5-dimethylthiazol-2-yl)-2,5-diphenyltetrazolium bromide) in a humidified CO_2 incubator at 37 °C for 4 hours. Cells were then lysed in SDS-HCl solution and incubated at 37 °C for 16 hours. The concentration of MTT formazan was measured by optical density (OD)

at 570 nm. Cell viability was calculated by dividing the OD of treated group by the mean OD of untreated controls.

Visualization of cerebral vessels

The cerebral vessels were mapped as described [16]. Higgins Black Magic waterproof ink (200–250 µl; Sanford Corp.) was injected into the left ventricle using a 26-gauge needle, and the right atrium opened to release the effluent. Mice were decapitated and the heads fixed in 10% neutral buffered formalin (Sigma-Aldrich) for 7 days. The brains were removed carefully from the skulls and imaged using a Leica EZ4HD stereomicroscope with integrated High Definition digital camera. The development of PcomA was graded on a qualitative scale of 0 to 3 [17]. Score 0 indicates no PcomA between anterior and posterior circulation; score 1 indicates PcomA in capillary phase; score 2 indicates small truncal PcomA; score 3 indicates truncal PcomA. To visualize the territory of MCA, we traced the peripheral branches of anterior cerebral artery (ACA) and MCA, and identified the points of anastomoses between ACA and MCA [3]. We connected the points of anastomoses to establish a "line of anastomoses". To assess the MCA territory, we measured the distances from the midline to the line of anastomoses at coronal planes 2, 4, and 6 mm from the frontal pole.

Supplemental Figures and Figure Legends

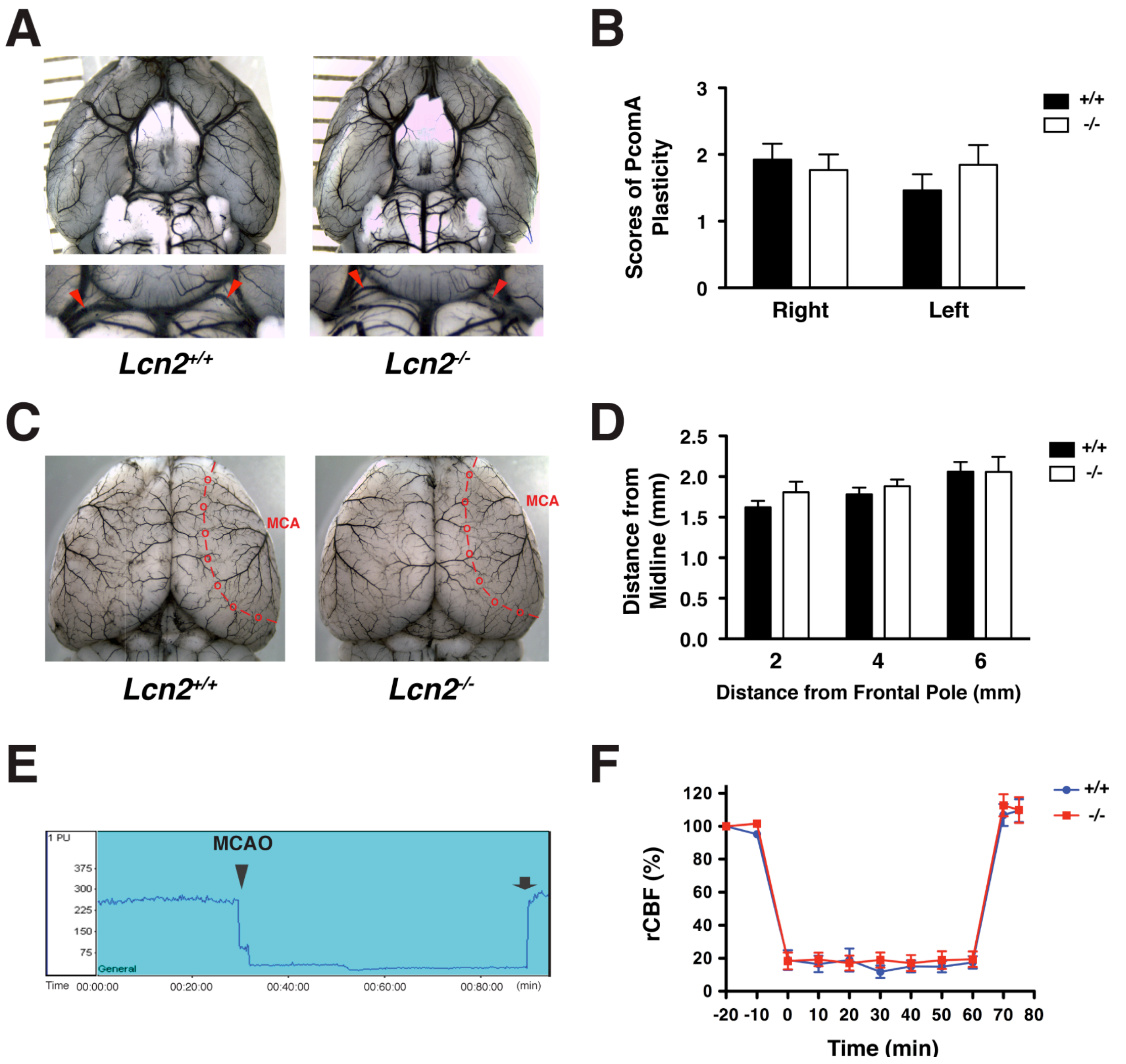

Supplemental Figure S1. Assessment of cerebrovascular anatomy and determination of regional cerebral blood flow (rCBF). (**A**) Shown are representative images of the brains from *Lcn2*$^{+/+}$ and *Lcn2*$^{-/-}$ mice perfused with black ink. The major arteries in the circle of Willis (upper panels) and PcomA (indicated by arrowheads in lower panels) were identified. (**B**) Shown are the scores of PcomA plasticity in *Lcn2*$^{+/+}$ (*n* = 13) and *Lcn2*$^{-/-}$ mice (*n* = 13). (**C**) Shown are the dorsal images of the brains from *Lcn2*$^{+/+}$ and *Lcn2*$^{-/-}$ mice perfused with black ink. The points of anastomoses were circled and connected to form the line of anastomoses. (**D**) Distances from the line of anastomoses to the midline in *Lcn2*$^{+/+}$ (*n* = 3) and *Lcn2*$^{-/-}$ (*n* = 3) mice were measured at coronal planes 2, 4, and 6 mm from the frontal pole. (**E**) Shown is a continuous tracing of Laser Doppler Flowmetry monitoring rCBF during one hour of tMCAO and reperfusion. Laser Doppler signal drops to ~12% of the baseline rCBF when the suture is advanced to the origin of MCA (arrow head), and returns back to the baseline when the suture is

withdrawn (arrow). (**F**) The rCBF during tMCAO was measured continuously in *Lcn2*$^{+/+}$ (n = 8) and *Lcn2*$^{-/-}$ (n = 8) mice. Steady-state rCBF before the MCAO were used as baseline (100%), and the subsequent changes after the onset of ischemia were shown as the percentage relative to the baseline. Time zero indicates the point of MCA occlusion. There were no significant differences in rCBF between *Lcn2*$^{+/+}$ and *Lcn2*$^{-/-}$ during tMCAO.

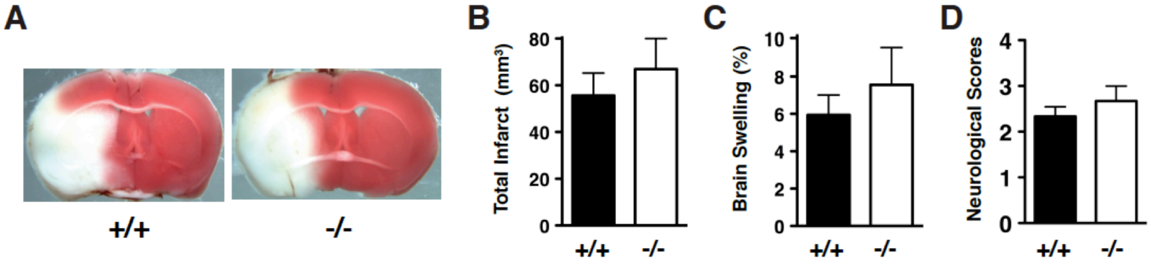

Supplemental Figure S2. Infarct size is similar in WT and LCN2 null mice after pMCAO. (**A**) Shown are representative images of TTC-stained brain slices after 24 hours of pMCAO from *Lcn2*$^{+/+}$ and *Lcn2*$^{-/-}$ mice. Total infarct volume (**B**), brain swelling (**C**), and neurological deficit scores (**D**) of *Lcn2*$^{+/+}$ (n = 8) and *Lcn2*$^{-/-}$ (n = 8) mice were measured after 24 hours of pMCAO.

Supplemental References

1. **Flo TH, Smith KD, Sato S, *et al.*** Lipocalin 2 mediates an innate immune response to bacterial infection by sequestrating iron. *Nature*. 2004; 432: 917-21.
2. **Longa EZ, Weinstein PR, Carlson S, *et al.*** Reversible middle cerebral artery occlusion without craniectomy in rats. *Stroke*. 1989; 20: 84-91.
3. **Chou WH, Choi DS, Zhang H, *et al.*** Neutrophil protein kinase Cdelta as a mediator of stroke-reperfusion injury. *J Clin Invest*. 2004; 114: 49-56.
4. **Chiang T, Messing RO, Chou WH.** Mouse model of middle cerebral artery occlusion. *J Vis Exp*. 2011.
5. **Prestigiacomo CJ, Kim SC, Connolly ES, Jr., *et al.*** CD18-mediated neutrophil recruitment contributes to the pathogenesis of reperfused but not nonreperfused stroke. *Stroke*. 1999; 30: 1110-7.
6. **Zhang L, Schallert T, Zhang ZG, *et al.*** A test for detecting long-term sensorimotor dysfunction in the mouse after focal cerebral ischemia. *J Neurosci Methods*. 2002; 117: 207-14.

7. **Thavasu PW, Longhurst S, Joel SP*, et al.*** Measuring cytokine levels in blood. Importance of anticoagulants, processing, and storage conditions. *J Immunol Methods*. 1992; 153: 115-24.
8. **Swanson RA, Morton MT, Tsao-Wu G*, et al.*** A semiautomated method for measuring brain infarct volume. *J Cereb Blood Flow Metab*. 1990; 10: 290-3.
9. **Lin TN, He YY, Wu G*, et al.*** Effect of brain edema on infarct volume in a focal cerebral ischemia model in rats. *Stroke*. 1993; 24: 117-21.
10. **Goslin K, Asmussen, A., and Banker, G.** Mass Cultures and Microislands of Neurons from Postnatal Rat Brain. 2nd ed. Cambridge, MA: The MIT Press; 1998.
11. **Qi ZH, Song M, Wallace MJ*, et al.*** Protein kinase C epsilon regulates gamma-aminobutyrate type A receptor sensitivity to ethanol and benzodiazepines through phosphorylation of gamma2 subunits. *J Biol Chem*. 2007; 282: 33052-63.
12. **Chou WH, Wang D, McMahon T*, et al.*** GABAA receptor trafficking is regulated by protein kinase C(epsilon) and the N-ethylmaleimide-sensitive factor. *J Neurosci*. 2010; 30: 13955-65.
13. **Jiang X, Mu D, Manabat C*, et al.*** Differential vulnerability of immature murine neurons to oxygen-glucose deprivation. *Exp Neurol*. 2004; 190: 224-32.
14. **Olive MF, McGeehan AJ, Kinder JR*, et al.*** The mGluR5 antagonist 6-methyl-2-(phenylethynyl)pyridine decreases ethanol consumption via a protein kinase C epsilon-dependent mechanism. *Mol Pharmacol*. 2005; 67: 349-55.
15. **Lowell CA, Berton G.** Resistance to endotoxic shock and reduced neutrophil migration in mice deficient for the Src-family kinases Hck and Fgr. *Proc Natl Acad Sci U S A*. 1998; 95: 7580-4.
16. **Panahian N, Maines MD.** Assessment of induction of biliverdin reductase in a mouse model of middle cerebral artery occlusion. *Brain Research Brain Research Protocols*. 2000; 6: 53-70.
17. **Murakami K, Kondo T, Kawase M*, et al.*** The development of a new mouse model of global ischemia: focus on the relationships between ischemia duration, anesthesia, cerebral vasculature, and neuronal injury following global ischemia in mice. *Brain Res*. 1998; 780: 304-10.
